# Influence of dietary coriander seeds and administration of *Lactobacillus acidophilus* on the performance of growing rabbits under subtropical climatic conditions

**DOI:** 10.17221/104/2024-VETMED

**Published:** 2025-09-29

**Authors:** Sherief Mohamed Abdel-Raheem, Mustafa Ahmed Kobeisy, Yasmin Adel Abdel-Wadood Gomaa, Ahmed Meligy Abdelghany Meligy, Mahmoud Elalfy, Mohsen Mohamed Farghaly

**Affiliations:** ^1^Department of Public Health, College of Veterinary Medicine, King Faisal University, Al-Hofuf, Al-Ahsa, Saudi Arabia; ^2^Department of Animal Production, Faculty of Agriculture, Assiut University, Assiut, Egypt; ^3^Department of Clinical Sciences, College of Veterinary Medicine, King Faisal University, Al-Hofuf, Al-Ahsa, Saudi Arabia

**Keywords:** coriander seeds, growth performance, intestinal morphology, microbiota modulation, probiotics

## Abstract

This study investigated the effects of coriander seed powder and *Lactobacillus acidophilus* solution (LAS) on the growth, nutrient digestibility, blood parameters, and intestinal health of growing rabbits under subtropical conditions. Forty Californian rabbits, aged 35 days with an average body weight of 588 ± 34 g, were randomly assigned to four groups: a control group fed a standard diet, a group (T1) receiving the basal diet supplemented with 1.5% coriander seed powder, a group (T2) receiving the basal diet with oral LAS at 1 × 10^9^ CFU/kg, and a group (T3) receiving both coriander seed powder and LAS. The study measured body weight, daily weight gain, feed conversion ratio, mortality rate, blood metabolites, nutrient digestibility, and intestinal histomorphology. Results showed that rabbits fed with 1.5% coriander seed powder had significant improvements in body weight gain, feed conversion, and a reduction in mortality compared to the control. Both coriander seed powder and LAS improved blood metabolites, nutrient digestibility, and intestinal health. However, the combination of both additives did not provide additional benefits over the individual treatments. The findings suggest that either 1.5% coriander seed powder or LAS can enhance growth performance and health in rabbits under subtropical conditions.

The early life of newly weaned rabbits is accompanied by several health issues, including increased body fat deposition associated with rapid growth, high incidence of metabolic disorders, and high mortality and morbidity (Ebeid et al. 2013).

Weaning rabbits face a significant risk of mortality and morbidity due to digestive disorders, resulting in substantial economic losses in industrial rabbit farms (Carabano et al. 2008). The shift from a milk-based diet to solid feed at weaning triggers a transformation in the rabbits’ digestive system. This change involves a transition from an exclusively endogenous hydrolytic system to one where caecum fermentation becomes active. Consequently, rabbits become highly susceptible to digestive disorders, especially if the introduction of solid food occurs earlier, facilitating the maturation of digestive physiology (Gidenne et al. 2009). To mitigate the economic impact of digestive diseases on rabbit farms, antibiotics are commonly introduced into the feed from the weaning stage up to eight weeks of age.

Initially, antimicrobial compounds were incorporated into animal feed at therapeutic doses primarily to treat and prevent infectious diseases. However, it did not take long for researchers to observe that antibiotics also had a positive effect on promoting growth. In recent times, global concern over antimicrobial resistance and the transfer of genes from animals to humans has been growing (Devirgiliis et al. 2013). Studies indicate that antibiotic-resistant *Escherichia coli* strains in humans may originate from poultry (Johnson et al. 2007). Since the ban on antibiotics as growth promoters in 2006, probiotics have emerged as potential alternatives for livestock production and health enhancement (Maertens et al. 2006).

Different options for antibiotics, including probiotics, prebiotics, bacteriocins, and herbal extracts, have shown promising results in preventing intestinal diseases and enhancing growth, meat quality, and immune responses in rabbits (Oso et al. 2013). The potential of herbs, spices, and various plant extracts as alternatives to antibiotics is currently being investigated, with some demonstrating growth-promoting effects, antimicrobial properties, stimulation of natural digestive enzymes, antioxidant properties, and other health benefits (Diaz-Sanchez et al. 2015).

Phytogenics, a novel group of feed additives, has been incorporated into poultry and animal diets to stimulate growth and improve health characteristics (Lee et al. 2004). These advantages arise from enhancements in gut health, including improvements in digestibility, alterations in digestive secretions, and positive effects on gut histology (Windisch et al. 2008). Additionally, certain phytobiotics contribute to the stability of the microbiome, reducing the presence of microbial toxins. This, in turn, lowers inflammation, allowing more protein to be directed toward growth rather than being utilised for the production of immune modulators (Prabakar et al. 2016). Several researchers have illustrated the antibacterial and antioxidant properties of coriander (Naeemasa et al. 2015).

Probiotics, live microbial supplements, foster a balanced gut environment in host animals, promoting health. Incorporating probiotics into diets is valued for enhancing growth rates, improving feed efficiency in rabbits, and positively modulating gut microbiota composition through the actions of beneficial microorganisms (Oso et al. 2013). Rabbit farming explores various probiotic sources, encompassing bacterial and yeast strains such as colonising (*Lactobacillus* and *Enterococcus* spp.) and non-colonising types (*Bacillus* spp*., Saccharomyces cerevisiae*). Bhatt et al. (2017) found that supplementing with probiotics, particularly *Lactobacillus acidophilus*, improved the digestibility and utilisation of nutrients, boosted body weight gain, and improved feed conversion ratios, with no significant changes observed in carcass traits, composition, and fatty acid profiles.

Previous studies investigating the use of coriander seeds (*Coriandrum sativum*) and *Lactobacillus acidophilus,* either alone or in combination, as growth promoters in growing rabbits are scarce. Therefore, the current research aims to explore the potential effectiveness of phytogenic compounds such as coriander and probiotics such as *Lactobacillus acidophilus,* either individually or in combination, on the growth performance, carcass traits, and nutrient digestibility of growing rabbits.

## MATERIAL AND METHODS

### Ethical approval

The experimental procedures of this study were conducted according to the standards set by the Egyptian Medical Research Ethics Committee (No. 14-126) and the Ethics Committee of Animal Experimentation at the Faculty of Agriculture, Assiut University.

### Animals, management, and diets

Forty weaned purebred Californian rabbits (not hybrid), obtained from a certified local breeding farm, clinically healthy and averaging 5 weeks of age with an average body weight of 588 ± 34 g, were randomly divided into four groups of ten rabbits each and observed over an 8-week experimental period. All animals were vaccinated against major rabbit diseases (viral haemorrhagic disease and myxomatosis) as per farm routine protocols. Prior to the experimental period (up to 35 days of age), rabbits received a commercial starter diet containing coccidiostats; after this age, experimental diets were coccidiostats-free. The control group received a basal diet, while the second group (T1) received the basal diet supplemented with 1.5% w/w coriander seed powder (mixed with the basal diet before pelleting). The third group (T2) received the basal diet along with oral administration of *Lactobacillus acidophilus* solution (LAS) at a dosage of 1 × 10^9^ CFU (colony-forming units)/kg of body weight via gavage. The fourth group (T3) received the basal diet containing 1.5% w/w coriander seeds alongside oral administration of LAS at a dosage of 1 × 10^9^ CFU/kg of body weight via gavage. The LAS was prepared using a commercial source of *Lactobacillus* (Biotic Balance, Pharma Care Europe Ltd., West Sussex, UK). Each ball from this product contains 1 billion live bacteria. One ball was ground and mixed, then dissolved in saline solution to prepare a solution at a concentration of 1 × 10^9^ CFU/ml. The solution was administered orally to the rabbits, which were individually housed in galvanised wire cages within an open-sided building. Each cage measured 40 × 40 × 35 cm in length, width, and height, equipped with a mesh floor. Each cage had a feeding box made from galvanised steel sheets, with automatic drinking nipples included. Rabbits had free access to feed and fresh water in each cage. Deworming was conducted before the experiment commenced. Rabbit manure was daily collected from the cages and promptly removed from the floor. The ingredients and nutrient contents of the experimental diets were analysed according to AOAC (2005) and presented in Table 1. All groups were housed under the same microclimatic conditions in an open-sided rabbitry, with temperatures ranging from 28 °C to 32 °C. All experimental conditions, including diet, housing, environmental temperature, and management practices, were uniformly applied across groups to ensure valid comparisons.

**Table 1:** Ingredients and chemical composition of the experimental diets

Ingredients (%)	Control	T1
Corn, grain	16.58	19.58
Soybean meal, 44%	19	19
Wheat bran	29	24.5
Berseem hay	30	30
Coriander seed powder	0	1.5
Molasses	3	3
Limestone	1	1
Dicalcium phosphate	0.5	0.5
Premix*	0.3	0.3
Common salt	0.3	0.3
dl- Methionine	0.3	0.3
Lysine	0.02	0.02
Total	100	100
Chemical composition (% unless stated)		
Dry matter	89.5	89.8
Digestible energy (kcal/kg DM)**	2 700	2 700
Crude protein	18.00	18.00
Crude fibre	11.35	11.25
Calcium	0.96	0.95
Phosphorus	0.71	0.66
Ether extract	2.67	2.71
Methionine + cysteine	0.78	0.77

The composition of ingredients and feed analysis is reported a feed basis

*The vitamin and mineral premix provided per kg of diet: vitamin A, 4 000 000 IU; vitamin D3, 667 000 IU; vitamin E 3 334 mg; vitamin K3, 1 167 mg; vitamin B1, 334 mg; vitamin B2, 1 667 mg; vitamin B3, 3 334 mg; B6, 500 mg; vitamin B12 33.4 mg, Folic acid, 334 mg; Biotin, 17 mg; Iron, 10 mg; Copper, 2.167 mg; Zinc, 18.334 mg; Manganese 20.0 mg; Iodine, 0.167 mg; Cobalt, 0.034 mg; Selenium, 0.034 mg; **Calculated based on NRC (1994) feed composition tables

At the beginning of the study and every week before morning feeding, the weights of the animals were recorded to determine their average daily weight gain. They received their pelleted diet once a day at 8:00 a.m., and the amount left was weighed to calculate their actual intake. The feed conversion ratio is calculated by comparing the grams of dry matter (DM) eaten to the grams of body weight gain. All animals were maintained under standardised management and hygiene protocols throughout the study. The daily mortality rates throughout the study were recorded and expressed as percentages.

### Gas Chromatography-Mass Spectrometry (GC-MS) analysis:

The coriander seed meal extract was analysed using a Shimadzu GCMS-QP2010 Plus instrument, Japan (Table 2). Helium gas, with an exceptionally high purity of 99.999 9%, served as the carrier gas at a flow rate of 2.25 ml/minute. The instrument was equipped with a DB-5MS capillary column (30 m long, 0.25 mm film thickness, 0.25 mm internal diameter). A 1 μl sample was injected in split mode (1 : 50) at an injection temperature of 260 °C. The GC-MS analysis started at an oven temperature of 70 °C, held for 7 min, then gradually increased by 7 °C per minute to reach 280 °C, where it was maintained for 10 minutes. The interface temperature was set to 220 °C, and the ion source to 250 °C. Data collection covered a range of 50–550 amu. The quantity of each component was estimated based on its peak area and expressed as a percentage of the total area of all peaks (El Sherif et al. 2020; Khattab et al. 2022).

**Table 2:** GC/MS chromatogram of coriander seed meal hexane extract

Component name	Height (%)	Peak height	Area (%)	Area	RT (minutes)
*P*-Cymene	0.21	12 062	0.27	36 717	6.958
Limonene	0.23	13 014	0.31	42 163	7.136
γ-Terpinen	1.08	60 933	1.41	190 528	8.302
α-pinene	0.02	1 384	0.01	1 808	8.879
Terpinolene	0.04	2 373	0.03	4 457	9.327
β-Linalool	24.05	1 358 167	30.39	4 110 318	9.99
Camphor, (1R,4R)-(+)-	0.98	55 129	1.27	172 455	11.503
1-Terpinen-4-ol	0.04	2 020	0.03	3 842	12.715
α-Terpineol	0.06	3 398	0.05	6 564	13.239
Palmitic acid, methyl ester	0.36	20 360	0.45	61 101	31.175
l-Ascorbic acid	1.69	95 291	2.26	306 311	31.859
Linoleic acid, methyl ester	3.21	181 348	3.9	527 989	34.278
Elaidic acid, methyl ester	8.7	491 363	12.03	1 628 209	34.435
*cis*-9,*cis*-12-Octadecadienoic acid	9.22	520 784	18.77	2 540 173	34.967
Octadec-9-enoic acid	48.09	2 714 974	26.39	3 570 834	35.168
Anethol	1.32	74 651	1.65	222 615	35.445
Geranyl acetate	0.24	13 288	0.21	28 827	38.295
Tetradecanal	0.16	9 213	0.16	21 740	40.212
Linalyl isobutyrate	0.3	17 124	0.41	55 979	41.462

RT = retention time

### Blood sampling

Blood samples (7 ml) were drawn from the marginal ear vein using sterile syringes after disinfecting the area with 70% ethanol.

The blood samples were handled with great care and separated into two portions. One portion was placed in tubes containing ethylenediamine tetraacetic acid (EDTA) for haematological analysis, while the other was stored for serum separation to assess biochemical indicators. After centrifugation at 805 *g* for 15 min, the resulting serum was meticulously collected and stored at –20 °C in Eppendorf tubes for further analysis. All haematological and serum biochemistry analyses were conducted following standard protocols, utilising an automatic, digital haematology analyser (Model Bc-3200, Shenzhen Mind ray Biomedical Electronics Co. Hamburg, Germany).

### Carcass characteristics

The carcass characteristics of rabbits from each group were examined at the end of the experiment. Before being slaughtered, they underwent a 12-hour fasting period with free access to fresh drinking water, and their live weight was recorded just before the slaughter. Immediately after bleeding, both edible and non-edible parts were documented. The dressing percentage, relative to the fasting body weight, was then calculated. The carcass preparation followed the procedures outlined by the World Rabbit Science Association, as detailed by Blasco and Ouhayoun (1996).

### Intestinal microbiota

Bacteria were isolated from caecal samples using the standard microbiological method, employing suitable dilutions in Ringer solution. The total counts of caecal bacteria were determined using count agar medium and MacConkey agar medium, as described by Zimbro et al. (2009), which utilised a serial dilution method to count coliform bacteria.

### Intestinal histomorphology

Following the slaughter process, the gastrointestinal tract (GIT) was divided into the duodenum and caecum, from which representative samples were collected. Tissue samples were fixed, embedded, and stained using standard protocols Bancroft and Stevens (1996). Using a light microscope, the stained tissue sections were examined, evaluating variables such as muscle and mucous membrane thickness, crypt width and depth, and villi height and width in both the duodenum and cecum. Imaging was conducted at ×500 and ×100 magnifications using an Axiostar microscope (Carl Zeiss, Oberkochen, Germany) connected to a computer equipped with Analysis-Opti Basic and soft imaging system software. Finally, the villus height/crypt depth ratio was calculated.

### Digestibility trials

Four trials were conducted to evaluate the digestibility and nutritional value of different experimental diets during the final week of the experiment, involving three rabbits from each group. The daily intake of pelleted feed during the faeces collection period was calculated by subtracting the residual feed from the initial amount offered. Diet samples were collected, ground through a 1-mm screen, and stored for subsequent chemical analysis. Faecal samples were systematically collected daily, following the European reference method for rabbit digestion trials, and frozen at –20 °C for chemical analysis. After drying and grinding, both feed and faecal samples underwent chemical analysis using AOAC (2005) methods. The apparent digestion coefficients for nutrients were determined by expressing the difference in nutrient content between consumed feed and faeces as a percentage of intake. Furthermore, the feeding value, represented by total digestible nutrient (TDN), was calculated based on the chemical analysis of ingredients and apparent digestibility coefficient, following the methodology of McDonald et al. (2010).

### Statistical analysis

The statistical analysis was performed using SAS v8.2 (2001; SAS Institute, Cary, NC, USA). The Shapiro-Wilk test was used to evaluate the data’s distribution for normality. Following confirmation of a normal distribution from this test, a one-way ANOVA was used to assess treatment effects on various variables. The significance between treatment means was determined using Tukey’s post-hoc test. Data were presented as mean ±SE, with significance set at *P* < 0.05.

## RESULTS

Supplementing the rabbit diet with 1.5% coriander seeds (T1) resulted in a significant enhancement (*P* = 0.049) in both average body weight gain and daily gain (BWG) compared to the control group (Table 3). However, no significant differences were observed among the treated groups. Although the daily feed intake of rabbits on both control and treated diets showed no significant effect, there was a trend for rabbits receiving either LAS alone or LAS with coriander seeds to exhibit reduced feed intake. The feed conversion ratio (FCR), expressed as g feed/g gain, was significantly improved (*P* = 0.010) in all treated groups of rabbits compared to the control group. Additionally, the inclusion of coriander seeds in the rabbit’s diet or the oral administration of LAS or both led to a reduction in the mortality rate to zero percent compared to the control group.

**Table 3:** Effect of dietary coriander seed, oral administration of LAS, or their combination on rabbit growth performance, feed intake, and feed conversion ratio

Item	Control	Treatment			*P*-value
		T1	T2	T3	
Initial body weight (g)	626 ± 26.6	582.5 ± 41.9	577 ± 32.8	567.5 ± 36.4	0.645
Final body weight (g)	2 287.5 ± 65.01	2 472.0 ± 75.5	2 378.5 ± 52.6	2 359 ± 79.5	0.318
Total BW gain (g)	1 661.5^b^ ± 55.9	1 889.5^a^ ± 74.9	1 801.5^ab^ ± 41.4	1 791.5^ab^ ± 68.8	0.048
Daily weight gain (g/day)	29.7^b^ ± 1.0	33.74^a^ ± 1.3	32.3^ab^ ± 0.7	31.8^ab^ ± 1.2	0.049
Feed intake (g/day)	104.9 ± 3.6	107.4 ± 3.8	97.6 ± 3.6	101.7 ± 2.8	0.227
Feed conversion ratio (g feed/g gain)	3.54^a^ ± 0.12	3.18^b^ ± 0.11	3.02^b^ ± 0.1	3.2^b^ ± 0.1	0.010
Mortality rate (%)	10	0	0	0	–

^a,b^Means of the same row in each item with different superscripts are significantly different (*P *< 0.05)

BW = body weight; CFU = colony-forming units; LAS = *Lactobacillus acidophilus* solution; T1 = 1.5% coriander seed; T2 = *Lactobacillus acidophilus* (1 × 10^9^ CFU/kg BW); T3 = coriander + *Lactobacillus acidophilus*

### Blood metabolites

The data from Table 4 reveal notable findings regarding blood constituents. The results show that rabbits administered LAS in the T2 group had significantly lower average albumin concentration in blood serum compared to other groups (*P* = 0.051). However, no significant differences were detected between the treatment and control groups for total protein, globulin, and glucose concentrations. Additionally, the serum concentrations of triglycerides, cholesterol, high-density lipoprotein cholesterol (HDL), and low-density lipoprotein cholesterol (LDL) were significantly lower (*P *< 0.05) in rabbits that received coriander seeds alone (T1) or in combination with LAS (T3) compared to the other groups. Furthermore, the total antioxidant capacity (TAC) of rabbits’ serum treated with either coriander seeds or LAS and their combination increased by approximately 20% compared to the control group.

**Table 4:** Effect of dietary coriander seed, oral administration of LAS or their combination on some blood metabolites of rabbits

Item	Control	Treatment			*P*- value
		T1	T2	T3	
Total protein (g l^–1^)	54.8 ± 2.2	55.2 ± 4.6	57.2 ± 3.4	51.4 ± 2.6	0.675
Albumin (g l^–1^)	31.8^a^ ± 0.6	32.8^a^ **± 0.7**	27.0^b^ ± 2.5	32.0^a^ ± 1.5	0.051
Globulin (g l^–1^)	23.0 ± 2.1	22.7 ± 4.8	28.5 ± 4.9	19.6 ± 2.6	0.461
Glucose (mg l^–1^)	1 018.0 ± 47.6	896.5 ± 63.5	1 009.8 **±** 49.4	972.0 ± 75.3	0.480
Triglycerides (mg l^–1^)	1 220.0^a^ ± 138.4	907.7^b^ ± 58.6	1 378.0^a^ ± 84.8	890.7^b^ ± 75.7	0.003
Cholesterol (mg l^–1^)	885.8^a^ ± 63.7	602.0^b^ ± 53.6	847.6^a^ ± 72.0	566.2^b^ ± 35.1	0.001
HDL-cholesterol (g l^–1^)	457.2^a^ ± 53.2	319.7^bc^ ± 38.0	412.8^ab^ ± 29.3	278.7^c^ ± 15.9	0.009
LDL-cholesterol (mg l^–1^)	203.8^a^ ± 36.5	87.0^b^ ± 13.4	146.3^ab^ ± 27.5	78.7^b^ ± 3.3	0.004
TAC (μmol l^–1^)	3.47 ± 0.13	4.39 ± 0.22	4.35 ± 0.34	4.31 ± 0.38	0.123

^a–c^Means of the same raw in each item with different superscripts are significantly different (*P* < 0.05)

BW = body weight; CFU = colony-forming units; HDL = high-density lipoprotein cholesterol; LAS = *Lactobacillus acidophilus* solution; LDL = low-density lipoprotein cholesterol; T1 = 1.5% coriander seed; T2 = *Lactobacillus acidophilus* (1 × 10^9^ CFU/kg BW); T3 = coriander *+ Lactobacillus acidophilus*; TAC = total antioxidant capacity

### Haematological variables

Supplementation of the diet with 1.5% coriander seed and the administration of LAS, either separately or in combination, resulted in elevated total white blood cell (WBC) counts compared with the control group, with increases of 17.6%, 14.2%, and 4.1%, respectively (Table 5). Notably, the eosinophil percentage was significantly higher in T2 compared to T3, while the lymphocyte percentage increased significantly in all treatment groups compared to the control group. Conversely, the percentage of neutrophils was lower in the treatment groups than in the control. Additionally, basophils increased with LAS administration alone in T2 when compared to other groups. In Table 6, no significant differences were observed in red blood cell (RBC) count, haemoglobin, haematocrit, mean corpuscular volume (MCV), or platelets between treatment and control groups. However, there was a notable decrease in mean corpuscular haemoglobin (MCH) with LAS administration alone in T2 in comparison with the control group, and the mean corpuscular haemoglobin concentration (MCHC) was lower in rabbits that received the combination of coriander seed and LAS (T3) in comparison with the control group. Additionally, mean platelet volume (MPV) was lower in all treatment groups compared to the control group.

**Table 5:** Effect of dietary coriander seed, oral administration of LAS, or their combination on haematological indicators of white blood cells of rabbits

Item	Control	Treatment			*P*- value
		T1	T2	T3	
WBC’s (×10^3^)	9.83 ± 1.23	11.93 ± 1.50	11.45 ± 0.99	10.25 ± 0.76	0.546
Eosinophil (%)	0.82^ab^ ± 0.07	0.52^ab^ ± 0.13	1.00^a^ ± 0.26	0.40^b^ ± 0.18	0.041
Lymphocytes (%)	45.01^b^ ± 0.76	63.47^a^ ± 3.07	61.98^a^ ± 1.64	61.42^a^ ± 2.88	0.001
Monocytes (%)	4.65 ± 0.18	4.67 ± 0.47	4.97 ± 0.64	4.01 ± 0.45	0.534
Neutrophils (%)	47.73^a^ ± 1.08	30.83^b^ ± 1.98	27.08^b^ ± 2.70	33.47^b^ ± 2.24	0.001
Basophiles (%)	1.30^b^ ± 0.09	1.43^b^ ± 0.08	2.32^a^ ± 0.24	1.23^b^ ± 0.19	0.001

^a,b^Means of the same raw in each item with different superscripts are significantly different (*P* < 0.05)

BW = body weight; CFU = colony-forming units; LAS = *Lactobacillus acidophilus* solution; T1 = 1.5% coriander seed; T2 = *Lactobacillus acidophilus* (1 × 10^9^ CFU/kg BW); T3 = coriander *+ Lactobacillus acidophilus*; WBC = white blood cells

**Table 6:** Effect of dietary coriander seed, oral administration of LAS, or their combination on haematological indicators of red blood cells of rabbits

Item (%)	Control	Treatment			*P*- value
		T1	T2	T3	
RBC’s (× 10^6^/microl)	5.80 ± 0.34	5.82 ± 0.21	6.02 ± 0.17	5.49 ± 0.08	0.408
Haemoglobin (g l^–1^)	116.3 ± 4.6	122.6 ± 3.1	122.5 ± 3.1	114.8 ± 0.6	0.211
Haematocrit (PCV) (%)	37.65 ± 1.26	40.02 ± 0.97	40.03 ± 0.96	37.45 ± 0.17	0.101
MCH (pg)	21.88^a^ ± 0.69	20.88^ab^ ± 0.30	20.47^b^ ± 0.27	20.90^ab^ ± 0.21	0.045
MCV(μm^3^)	69.27 ± 2.321	68.00 ± 0.94	66.75 ± 1.14	68.50 ± 0.62	0.620
MCHC (g l^–1^)	317.1^a^ ± 6.5	307.5^ab^ ± 0.1	307.0^ab^ ± 0.7	306.3^b^ ± 0.6	0.042
Platelets (mg l^–1^)	3 755.0 ± 199.8	3 730.0 ± 145.7	3 991.7 ± 1258	3 480.0 ± 353.1	0.474
MPV (μm^3^)	8.87^a^ ± 0.11	8.10^b^ ± 0.23	8.02^b^ ± 0.25	7.55^b^ ± 0.14	0.001

^a,b^Means of the same raw in each item with different superscripts are significantly different (*P *< 0.05)

BW = body weight; CFU = colony-forming units; haematocrit (PCV) = packed cell volume; LAS = *Lactobacillus acidophilus* solution; MCV = mean corpuscular volume; MCH = mean corpuscular haemoglobin; MCHC = mean corpuscular haemoglobin concentration; MPV = mean platelet volume; RBC = red blood cell; T1 = 1.5% coriander seed; T2 = *Lactobacillus acidophilus* (1 × 10^9^ CFU/kg BW); T3 = coriander *+ Lactobacillus acidophilus*

### Carcass traits

The results presented in Table 7 indicate that there were no significant differences (*P* < 0.05) in the hot carcass weight and internal organs between the control and treated groups, except for the pelt. Notably, the pelt was significantly higher (*P* < 0.05) in rabbits supplemented with 1.5% coriander seed alone compared with the control group. Furthermore, the dressing percentage was notably higher (*P* = 0.05) in rabbits administered only LAS (T2) compared to the control group.

**Table 7:** Effect of dietary coriander seed, oral administration of LAS, or their combination on hot carcass, an edible and non-edible part

Item	Control	Treatment			*P*-value
		T1	T2	T3	
Slaughter weight (g)	2 555.0 ± 45.37	2 666.67 ± 70.13	2 550.00 ± 90.44	2 473.33 ± 130.43	0.314
Hot carcass weight (g)	1 464.00 ± 20.45	1 566.67 ± 54.32	1 525.00 ± 50.91	1 439.17 ± 86.39	0.256
Dressing percentage (%)	57.30^b^ ± 0.86	58.75^ab^ ± 0.75	59.80^a^ ± 0.47	58.19^ab^ ± 0.61	0.050
Head (g)	140.17 ± 4.52	141.83 ± 1.56	135.33 ± 4.50	139.17 ± 4.38	0.832
Kidney (g)	21.00 ± 2.40	20.17 ± 0.87	17.50 ± 1.06	19.67 ± 2.04	0.531
Liver (g)	69.67± 4.30	71.83 ± 3.11	70.00 ± 3.84	61.17 ± 1.72	0.149
Heart and lungs (g)	27.00 ± 1.55	28.33 ± 0.56	24.17 ± 1.66	25.00 ± 2.51	0.337
Pelt (g)	413.33^b^ ± 17.38	471.83^a^ ± 12.88	441.33^ab^ ± 13.28	428.00^ab^ ± 23.54	0.048
Intestine (g)	349.33 ± 21.01	369.83 ± 17.11	252.50 ± 21.80	351.00 ± 17.80	0.981

^a,b^Means of the same raw in each item with different superscripts are significantly different (*P *< 0.05)

BW = body weight; CFU = colony-forming units; LAS = *Lactobacillus acidophilus* solution; T1 = 1.5% coriander seed; T2 = *Lactobacillus acidophilus* (1 × 10^9^ CFU/kg BW); T3 = coriander + *Lactobacillus acidophilus*

### Nutrient digestibility and feeding values

The data presented in Table 8 indicate that the group supplemented with coriander seeds (T1) has shown the highest levels of digestibility for both dry matter (DM) and organic matter (OM), significantly exceeding (*P* < 0.05) all other groups. In both the T1 and T2 groups, there was a significant increase (*P* < 0.001) in crude protein digestibility in comparison with the control and T3 groups, with values of 51.52 and 48.72 vs 38.28 and 42.19, respectively. However, ether extract digestibility was notably lower (*P* < 0.05) in the T1 and T2 groups in comparison with the control and T3 groups. Moreover, crude fibre digestibility was lower (*P* < 0.05) in all treated groups in comparison with the control. Interestingly, the T1 and control groups exhibited a higher (*P* < 0.05) feeding value, expressed as total digestible nutrients (TDN), compared with T2, with values of 38.72 and 38.07 vs 34.9, respectively.

**Table 8:** Effect of dietary coriander seed, oral administration of LAS, or their combination on nutrient digestibility and diet feeding value of rabbits

Item (%)	Control	Treatment			*P-* value
		T1	T2	T3	
Nutrient digestibility (%)					
DM	37.63^b^ ± 1.96	45.60^a^ ± 1.52	36.28^b^ ± 1.33	35.35^b^ ± 0.56	0.003
OM	40.06^b^ ± 1.69	47.47^a^ ± 1.13	39.63^b^ ± 0.94	38.42^b^ ± 0.05	0.001
CP	38.28^b^ ± 1.52	51.52^a^ ± 0.30	48.72^a^ ± 1.69	42.19^b^ ± 2.14	0.001
CF	41.62^a^ ± 1.75	35.82^b^ ± 0.74	22.85^d^ ± 0.54	27.16^c^ ± 0.49	0.001
EE	71.22^a^ ± 0.05	58.58^b^ ± 1.64	50.35^c^ ± 2.85	65.90^a^ ± 1.14	0.001
NFE	38.83 ± 0.73	41.78 ± 1.76	41.46 ± 1.63	39.74 ± 0.41	0.362
Feeding value (%)					
TDN	38.07^a^ ± 1.04	38.72^a^ ± 1.04	34.90^b^ ± 0.72	36.45^ab^ ± 0.59	0.032

^a–c^Means of the same raw in each item with different superscripts are significantly different (*P* < 0.05)

BW = body weight; CF = crude fibre; CFU = colony-forming units; CP = crude protein; DM = dry matter; EE = ether extract; LAS = *Lactobacillus acidophilus* solution; NFE = nitrogen-free extract; OM = organic matter; T1 = 1.5% coriander seed; T2 = *Lactobacillus acidophilus* (1 × 10^9^ CFU/kg BW); T3 = coriander + *Lactobacillus acidophilus*; TDN = total digestible nutrients

### Intestinal microbiota

Table 9 outlines the impact of coriander seeds (T1), LAS (T2), or their combination (T3) on the total bacterial and coliform count in the cecum of rabbits. The results show that there were no significant differences in the bacterial count between the control group and the groups that received the different treatments. However, it is worth mentioning that the supplementation of coriander seeds led to a numerical increase of approximately 9.57% in the total bacterial count, while the administration of LAS resulted in a numerical increase of about 14.76% compared to the control group.

**Table 9:** Effect of dietary coriander seed, oral administration of LAS, or their combination on total bacterial and coliform count in the cecum of growing rabbits

Item	Control	Treatment			*P*-value
		T1	T2	T3	
Total bacterial count (CFU/g × 10^9^)	3.87 ± 0.37	4.28 ± 0.37	4.54 ± 0.12	3.87 ± 0.37	0.430
Total coliform count (CFU/g × 10^9^)	4.04 ± 0.33	4.31 ± 0.38	4.36 ± 0.17	4.23 ± 0.17	0.863

BW = body weight; CFU = colony-forming units; LAS = *Lactobacillus acidophilus* solution; T1 = 1.5% coriander seed; T2 = *Lactobacillus acidophilus* (1 × 10^9^ CFU/kg BW); T3 = coriander + *Lactobacillus acidophilus*

### Intestinal histomorphology

**Figure 1:**
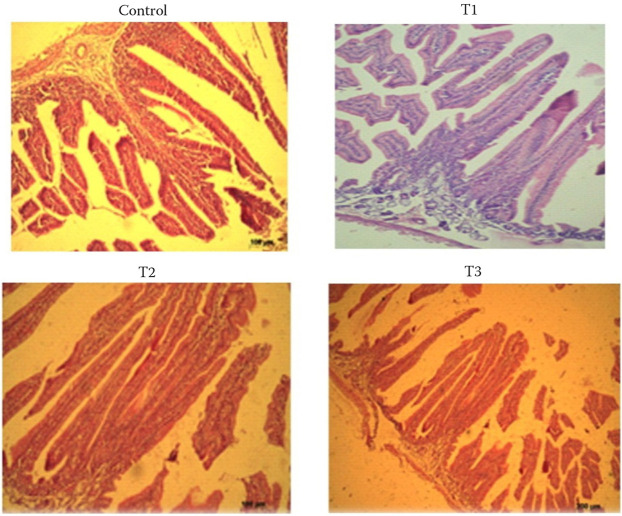
Duodenum of rabbits showing normal intestinal villi (bar = 100 μm; H&E stain) BW = body weight; CFU = colony-forming units; T1 = 1.5% coriander seed; T2 = *Lactobacillus acidophilus* (1 × 10^9^ CFU/kg BW); T3 = coriander + *Lactobacillus acidophilus*

**Figure 2:**
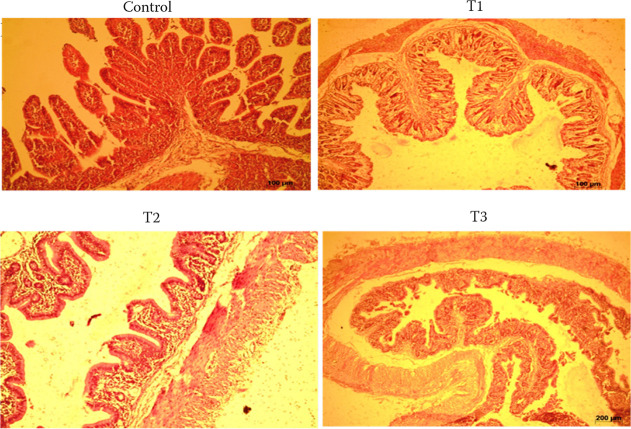
Caecum of rabbit showing mucosa (bar = 100 μm; H&E stain) BW = body weight; CFU = colony-forming units; T1 = 1.5% coriander seed; T2 = *Lactobacillus acidophilus* (1 × 10^9^ CFU/kg BW); T3 = coriander + *Lactobacillus acidophilus*

The data in Table 10, Figures 1 and 2 illustrate a significant reduction (*P* < 0.05) in the length of duodenal villi in rabbits orally administered LAS solution alone (T2) or in combination with coriander seeds (T3), compared to both T1 and the control groups. Conversely, the depth of duodenal crypts was notably higher (*P* < 0.05) in rabbits from T1, given coriander seed supplementation alone, compared to the control group (223.02 vs. 139.81 μM), with no significant differences among the treated groups. Additionally, a significant decrease (*P* = 0.003), in the length of large intestinal folds was observed in the group of rabbits receiving the combination of coriander seeds and *Lactobacillus acidophilus* (T3) compared to the other groups.

**Table 10:** Effect of dietary coriander seed, oral administration of LAS, or their combination on histological indicators of the rabbit intestine

Item	Control	Treatment			*P*- value
		T1	T2	T3	
Length of duodenal villi (μm)	1 057.20^a^ ± 42.25	1 010.58^a^ ± 41.49	660.65^b^ ±74.23	800.39^b^ ± 46.55	0.001
Depth of duodenal crypts (μm)	1 39.81^b^ ± 10.30	223.02^a^ ± 18.22	163.26^ab^ ± 22.46	162.22^ab^ ± 29.6	0.050
Length of large intestinal folds (μm)	815.46^a^ ± 62.31	823.58^a^ ± 15.73	748.38^a^ ± 47.72	480.33^b^ ± 55.27	0.003

^a,b^Means of the same raw in each item with different superscripts are significantly different (*P *< 0.05)

BW = body weight; CFU = colony-forming units; LAS = *Lactobacillus acidophilus* solution; T1 = 1.5% coriander seed; T2 = *Lactobacillus acidophilus* (1 × 10^9^ CFU/kg BW); T3 = coriander + *Lactobacillus acidophilus*

## DISCUSSION

### Growth performance of growing rabbits

Coriander (*Coriandrum sativum*) has long been recognised in traditional medicine for its broad spectrum of health-promoting properties, including antifungal, antioxidant, hypolipidemic, antimicrobial, hypocholesterolaemic, and anticonvulsant activities. The enhanced weight gain observed in rabbits supplemented with coriander may be primarily attributed to its bioactive constituents, particularly essential oils and unsaturated fatty acids (Table 2). This improvement in growth performance is likely not solely due to the plant’s antioxidant capacity, but also to its ability to stimulate digestive enzyme secretion, reinforce gut barrier integrity, and modulate the intestinal microbiota. Among its key constituents, linalool – a major component of essential oil – has demonstrated potent antioxidant, anti-inflammatory, and immunostimulatory effects, all of which contribute to improved health and growth outcomes. Furthermore, the presence of monounsaturated and polyunsaturated fatty acids in coriander enhances the antioxidant defence system and mitigates inflammation, thereby supporting metabolic efficiency and overall physiological performance (Hosseinzadeh et al. 2014). These bioactive compounds collectively foster an improved metabolic environment, promoting weight gain in rabbits. These active ingredients not only improve feed intake due to their appetising effects but also exhibit antimicrobial properties, contributing to a prebiotic effect by inhibiting pathogenic and saprophytic microorganisms (Mohammed et al. 2018). Conversely, the administration of *Lactobacillus acidophilus*, either alone (T2) or in combination with coriander seed supplementation (T3), has been linked to significant improvements in average body weight gain and daily gain in growing rabbits. This improvement is likely due to enhanced gut health through the reduction of pathogenic bacteria, an increase in beneficial flora, and an improved immune system despite limited data on probiotic use in rabbits compared to other species such as pigs and poultry (Copeland et al. 2009). Notably, such probiotic administration does not significantly affect feed intake, in agreement with previous studies (Oso et al. 2013). However, these interventions result in a higher growth rate and improved feed conversion ratio (FCR) by enhancing feed digestion and absorption. The recorded mortality rate in the control group (10%) was likely attributable to heat stress and subclinical coccidial infection, as inferred from clinical signs and post-mortem findings. In contrast, the absence of mortality in the treatment groups suggests a protective role of *Coriandrum sativum* and *Lactobacillus acidophilus* in promoting gut health and enhancing resilience to environmental stressors. This protective effect may be mediated through improved intestinal morphology and a favourable shift in gut microbiota composition, as reported by Oso et al. (2013) and Adli et al. (2023). Alagbe (2018) similarly observed reduced mortality and enhanced immune response in quails supplemented with coriander leaf meal. Furthermore, the probiotic-related reduction in mortality among growing rabbits reported by Amber et al. (2014) reinforces the synergistic benefits of phytogenic additives and probiotics in enhancing growth performance, feed utilisation, and survival rates. The antimicrobial activity of coriander, coupled with the pathogen-inhibitory action of *L. acidophilus* via competitive exclusion, likely contributed to the improved health outcomes observed in the treated groups, consistent with earlier findings in poultry (Hosseinzadeh et al. 2014).

When combined with coriander seed and *Lactobacillus acidophilus*, a probiotic known for its immunomodulatory functions and production of short-chain fatty acids (SCFAs), a synergistic effect is likely to occur. *L. acidophilus* contributes to gut health by enhancing the intestinal barrier, modulating immune responses, and supporting microbial balance through SCFA production such as butyrate and propionate (Ratajczak et al. 2019). Together, these additives may improve metabolic performance, enhance immune function, and promote intestinal health under challenging environmental conditions.

### Blood constituents

The slight reduction in serum albumin levels observed following probiotic supplementation in the experimental groups may be attributed to enhanced protein turnover and utilisation in physiological processes such as mucosal immunity and tissue repair. Probiotics have been shown to modulate the immune system, promoting the proliferation of regulatory T cells and influencing cytokine production, which are essential for maintaining immune homeostasis and facilitating tissue repair mechanisms (Guo and Lv 2023). Furthermore, probiotics can enhance the integrity of the intestinal barrier by strengthening tight junction proteins and promoting mucin secretion, thereby supporting mucosal immunity. These activities may increase the demand for amino acids and proteins, potentially leading to a slight decrease in serum albumin levels as proteins are utilised for immune responses and tissue maintenance (Virk et al. 2024). The supplementation of coriander seed has been observed to lead to a decrease in blood serum glucose concentrations by 13.6% compared to control groups, a phenomenon that may be attributed to the essential oil content in coriander seeds, particularly known for its hypoglycaemic effect (Aissaoui et al. 2011). This effect is possibly due to insulin-like substances within coriander seeds that stimulate β cells for higher insulin production, thereby enhancing glucose metabolism and potentially regenerating pancreatic tissue (Gholamali Jelodar et al. 2007). The impact of coriander seed on lipid profiles, either alone or in combination with *Lactobacillus acidophilus*, mirrors the findings of previous studies that document its hypolipidemic effects, possibly due to components such as linalool oil (Mohammed et al. 2018). The reduction in HDL cholesterol observed in the experimental groups remained within normal physiological ranges and may indicate alterations in lipid metabolism due to feed additives. This could be interpreted as part of a broader metabolic shift induced by coriander or probiotic intake. Jakulj et al. (2005) explained that coriander supplementation reduces cholesterol and phospholipid levels by interfering with the absorption of dietary fats and promoting excretion of cholesterol. Studies by Ooi and Liong (2010) have shown that rabbits receiving a combination of *Lactobacillus acidophilus* and coriander seed in their diets had reduced serum cholesterol and triglyceride concentrations.

### Haematological parameters

The changes observed in haematological parameters across the experimental groups are likely attributable to the immunostimulatory effects of the dietary additives. The elevated lymphocyte counts may reflect an enhancement in immune competence, whereas the stability of red blood cell indices indicates the preservation of systemic health and physiological balance. Alterations in total white blood cell (WBC) count and differential cell profiles remained within normal physiological limits, suggesting a beneficial modulation of immune function. These findings imply that the observed haematological responses are indicative of improved immune efficiency rather than pathological changes. Consistent with these observations, previous studies by Taha et al. (2019) and Alagbe (2018) reported that dietary inclusion of coriander seed exerts no adverse effects on haematological parameters, further supporting the safety and potential health-promoting effects of such phytogenic feed additives.

Mohammed et al. (2018) attributed the increase in WBC count observed in lambs receiving coriander powder to the sterol and tocopherol compounds present in coriander seeds, known for their immunomodulatory effects and antioxidant properties. These compounds contribute to immune response enhancement by suppressing free radicals. Similarly, the influence of *Lactobacillus acidophilus* on haematological parameters aligns with findings by Bovera et al. (2012) demonstrating notable differences in cell profiles between groups receiving *Lactobacillus* and control groups, with increased lymphocyte percentages and decreased neutrophils and eosinophils. Elevated lymphocytes in treated groups suggest immune activation via coriander’s immunomodulatory terpenes and *L. acidophilus*-induced cytokine production (Omar et al. 2024). Neutrophil reductions align with decreased inflammation, consistent with coriander’s anti-inflammatory effects. Although both coriander seed alone or in combination with LAS fail to induce significant effect on total WBC or RBC count, Salahuddin et al. (2013) reported that the positive effect of probiotics, showing significant increases in red blood cell count and haemoglobin concentration with higher probiotic concentrations. Additionally, Abdelhady and El-Abasy (2015) highlight the immunomodulatory effects of probiotic blends, indicating significant enhancements in phagocytic activity, phagocytic index, and total leukocyte count in rabbits infected with *Pasteurella multocida*. These findings collectively underscore the potential of coriander seed and *Lactobacillus acidophilus* as dietary supplements in promoting immune function and overall health in livestock and experimental animals.

### Carcass traits

The current findings indicate that the oral administration of *Lactobacillus acidophilus* to growing rabbits enhanced the dressing percentage without causing significant effects on internal organs. These outcomes are consistent with earlier research (Fathi et al. 2017). Conversely, several researchers have reported no significant impact of probiotics on carcass traits in rabbits, as observed in studies by Bhatt et al. (2017).

### Nutrient digestibility and feeding value of diets

The supplementation of rabbit diets with 1.5% coriander seeds and oral administration of *Lactobacillus acidophilus* have been shown to enhance the digestibility of dry matter (DM), organic matter (OM), and crude protein (CP). This improvement in digestibility may be attributed to the essential oil content of coriander seeds, which has been demonstrated to enhance nutrient digestibility (Hernandez et al. 2004). Additionally, coriander’s appetising effects, as suggested by Windisch et al. (2008), may stimulate enzyme secretion and digestive juice production, thereby promoting digestion and peristaltic motion. Oral administration of *Lactobacillus acidophilus* may further enhance crude protein digestibility by positively impacting gastrointestinal health and stimulating host enzyme production, as indicated by Mateos et al. (2010). Furthermore, studies by Giang et al. (2010) in piglets and Bhatt et al. (2017) in Chinchilla rabbits have demonstrated that the inclusion of probiotics in diets improves nutrient digestibility. These findings collectively suggest that the enhanced digestibility observed with coriander seed supplementation and oral administration of *Lactobacillus acidophilus* could be attributed to their beneficial effects on digestive processes and gut health.

### Intestinal microbiota

The supplementation of coriander seeds and administration of *Lactobacillus acidophilus* solution did not significantly affect the total bacterial and coliform count in the caecum digesta of rabbits, consistent with findings by Gidenne et al. (2009), which suggested minimal changes in the rabbit caecum microflora with age. Molecular microbiological techniques employed by Abecia et al. (2005) highlighted bacteria as the main constituents in the rabbit caecum, with a notable absence of Lactobacilli due to the highly acidic stomach environment (Cheeke 1987). Similarly, Esteghamat (2014) observed no impact on *E. coli* count with different percentages of coriander seeds in Japanese quail diets, while Carson et al. (2002) emphasised coriander seed essential oils’ antimicrobial properties, disrupting bacterial cell walls and eliminating pathogenic bacteria through hydrophobic interactions (Duarte et al. 2016). Phuoc and Jamikon (2017) demonstrated that *L. acidophilus* supplementation, alone or in combination with *B*. *subtilis*, could increase beneficial gut bacteria populations and decrease coliform populations in rabbits. These findings collectively suggest that while coriander seed and *Lactobacillus acidophilus* interventions may not significantly alter total bacterial and coliform counts in rabbit caecum digesta, they could influence the balance of beneficial and pathogenic bacteria, contributing to gut health.

### Intestinal histomorphology

Alterations in the morphological characteristics of the small intestine, including villus length, crypt depth, and the overall length of the intestine, play a pivotal role in nutrient absorption and digestive efficiency, with villus length and crypt depth being critical indicators of intestinal function and health (Makovicky et al. 2014). Recent studies have explored the effects of dietary supplements such as coriander seeds and the probiotic *Lactobacillus acidophilus* on these intestinal features. Notably, supplementation with 1.5% coriander seeds or the oral administration of *Lactobacillus acidophilus*, individually or in combination, has been shown to significantly increase the depth of duodenal crypts in rabbits, with coriander seed supplementation leading to a notable increase in crypt depth by approximately 60%, suggesting an enhanced mucosal mitotic index and improved microbial fermentation (Table 8). This is further supported by improvements in the digestibility of dry matter (DM) and organic matter (OM) in rabbits fed diets supplemented with coriander seeds (Table 7), indicating a positive impact on nutrient absorption and digestion efficiency. The beneficial effects of coriander seeds on gut morphology, attributed to their antimicrobial properties and potential to improve the regenerative capacity of epithelial cells, align with findings from studies on broiler diets, where the inclusion of coriander seeds resulted in increased villi length and crypt depth, thereby enhancing intestinal absorptive capacity (Ghazanfari et al. 2015). However, the impact of *Lactobacillus acidophilus* on gut histopathology, especially concerning the height of duodenal villi, has produced mixed results, with some studies observing no significant improvements compared to control groups (Hidayat et al. 2018). The addition of probiotics and coriander seed powder could potentially inhibit the proliferation of a wide array of pathogenic and non-pathogenic intestinal bacteria. This inhibition may lead to a decrease in intestinal colonisation and infection, ultimately reducing inflammation in the intestinal mucosa. Such a reduction could result in enhanced villus height and width, thereby boosting the secretory functions, digestion, and nutrient absorption processes. This overall improvement could significantly contribute to the maturation of the gut.

In conclusion, supplementing rabbit diets with either 1.5% coriander seed powder or *Lactobacillus acidophilus* improved growth performance, feed conversion, and health indicators, including nutrient digestibility and blood metabolites. While both treatments enhanced rabbit welfare, the individual use of coriander seeds showed the most significant benefits. The combination of both additives did not offer additional advantages over their individual inclusion.

While this study focused on growth performance and haematobiochemical indicators, we acknowledge the importance of microbiome composition and SCFA production in gut health. Future studies should include metagenomic and metabolomic profiling to capture these aspects.
